# One-Step Mask-Based Diffraction Lithography for the Fabrication of 3D Suspended Structures

**DOI:** 10.1186/s11671-018-2817-6

**Published:** 2018-12-05

**Authors:** Xianhua Tan, Tielin Shi, Jianbin Lin, Bo Sun, Zirong Tang, Guanglan Liao

**Affiliations:** State Key Lab of Digital Manufacturing Equipment and Technology, Huazhong University of Science and Technology, Wuhan, 430074 People’s Republic of China

**Keywords:** Three-dimensional suspended structure, Diffraction, Carbon microelectromechanical systems, Pyrolytic carbon structures

## Abstract

We propose a novel one-step exposure method for fabricating three-dimensional (3D) suspended structures, utilizing the diffraction of mask patterns with small line width. An optical model of the exposure process is built, and the 3D light intensity distribution in the photoresist is calculated based on Fresnel-Kirchhoff diffraction formulation. Several 3D suspended photoresist structures have been achieved, such as beams, meshes, word patterns, and multilayer structures. After the pyrolysis of SU-8 structures, suspended and free-standing 3D carbon structures are further obtained, which show great potential in the application of transparent electrode, semitransparent solar cells, and energy storage devices.

## Introduction

3D carbon microelectromechanical system (C-MEMS) structures have drawn more and more attentions owing to their excellent chemical stability, electrochemical activity, and biocompatibility [1–5]. Suspended carbon structures are the typical 3D C-MEMS structures free of any intermolecularity [2], presenting significant advantages in sensors [6, 7], microelectrodes [8, 9], and energy storage applications [9]. Various C-MEMS microstructures have been achieved through pyrolysis of polymer, in which SU-8 is the most widely used precursor for pyrolytic carbon structures [10, 11]. With respect to its low light absorption, it is easy to fabricate high aspect ratio microstructures with SU-8 [12]. However, it is still a great challenge to obtain suspended polymer template.

Diverse approaches have been developed to fabricate suspended microstructures, such as E-beam writer [13–15], X-ray [10, 16], and two-photon lithography [17–19]. Two-photon lithography is a feasible way for achieving complex suspended structures, such as suspended hollow microtubes, with great accuracy but low efficiency [17]. Taking the efficiency and cost into account, UV lithography could be a better choice for fabricating photoresist precursor. Multi-step lithography process with controlled exposure dose for fabricating suspended structures has been demonstrated [3, 6, 7, 20]. Lim et al. [21] fabricated suspended nanowires and nanomeshes using a two-step UV lithography process and obtained glassy carbon nanostructures through a pyrolysis process. Some one-step lithography methods have also been proposed. No et al. [22] achieved suspended microstructures by a single exposure process, during which an optical diffuser film was put on the Cr-masks. The diffuser film had a significant impact on the exposure process, leading to the deformation of photoresist patterns. Long et al. [2] successfully fabricated 3D suspended structures by controlling the exposure dose and air gap between the photoresist and photomask during the proximity exposure process, whereas the proximity exposure mode limited the fabricating resolution. Grayscale photolithography has also been applied in fabricating suspended structures with grayscale masks or maskless lithography systems [11, 23]. Since SU-8 is almost transparent when the light wavelength is above 350 nm [12], it is very difficult to control the accuracy of the thickness of the suspended layer by adjusting the exposure dose [8, 10]. Hemanth et al. [10] optimized the UV wavelength in the exposure process according to the properties of SU-8. They chose the UV wavelength of 405 nm for the high ratio microstructures and 313 nm for the suspended layer. However, the combination of exposure with different UV light wavelengths increases the costs and difficulties of the whole fabrication process.

In this study, we demonstrate a novel one-step mask-based diffraction lithography process that is compatible with most kinds of photoresist to fabricate 3D suspended structures. A 3D light intensity distribution is simulated in the photoresist according to Kirchhoff’s diffraction theory and further verified by experiments. The thickness of the suspended structures is controlled by the width of the patterns, and the suspended beams are broadened by stacking several line patterns side by side with proper spacing. Complex 3D suspended structures, such as beams with gradient thickness and full suspended meshes with word patterns, can be achieved by the one-step lithography process. Finally, the suspended carbon beams, meshes, and free-standing carbon meshes have also been obtained via a pyrolysis process.

## Methods and Experiments

### Optical Model of Diffraction Lithography

**Fig. 1:**
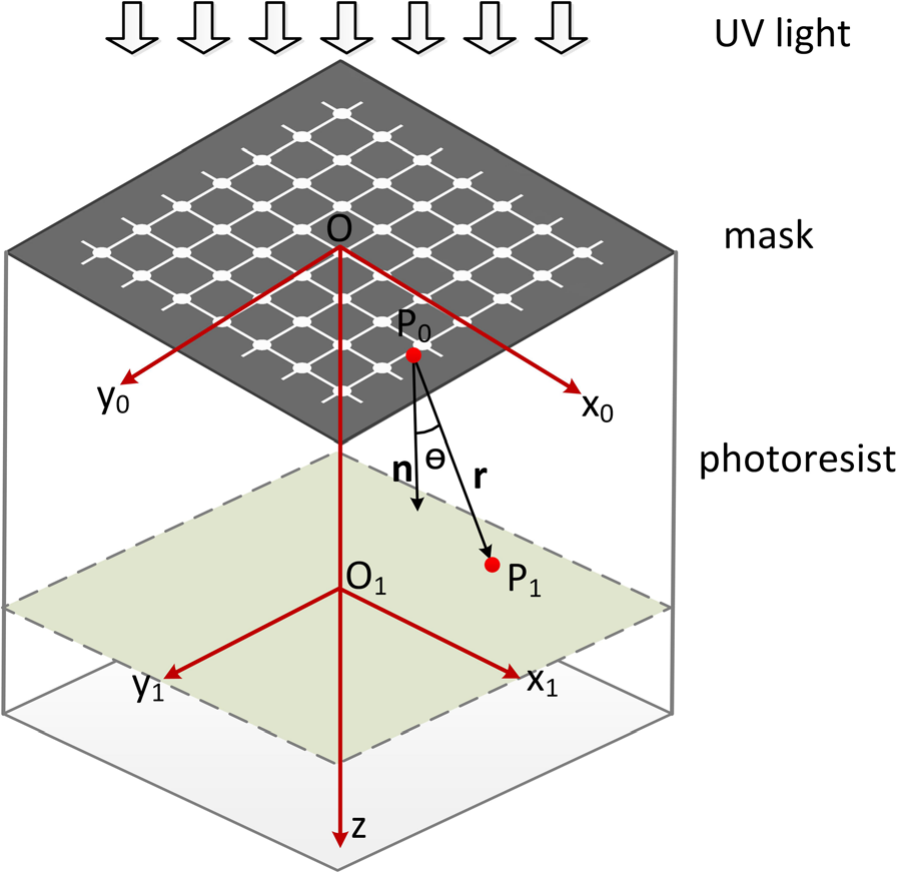
The optical model of the diffraction lithography

During the UV lithography process, the diffraction phenomenon will be very obvious when the pattern size is too small. Here, we utilize the diffraction of narrow patterns with several wavelength widths to fabricate suspended beams. In order to analyze the spatial light intensity distribution in the photoresist, we build an optical model (Fig. 1) for diffraction lithography based on Fresnel diffraction. The air gap between the photoresist and photo mask can be ignored since the exposure is carried out in a hard contact mode. The mask is illuminated with a plane wave at a typical wavelength of 365 nm, and the photoresist is treated as a transparent material with refractive index of 1.659 (the refractive index of SU-8 at 365 nm, measured by an ellipsometer). *P*_0_ is a point on the mask with a coordinate of (*x*_0_, *y*_0_, 0), and *P*_1_ is an arbitrary point in the photoresist with a coordinate of (*x*_1_, *y*_1_, *z*_1_).

According to the Fresnel-Kirchhoff diffraction formulation [24], the amplitude at point *P*_1_ in the photoresist is

1 $$ E\left({P}_1\right)=\frac{1}{2 j\lambda}\underset{\sum }{\iint }E\left({P}_0\right)\frac{\exp (jkr)}{r}\left(1+\cos \theta \right) ds $$

where *k* = 2*π*/*λ*, *λ* represents the wavelength of UV light in the photoresist, *E*(*P*_0_) is the light wave amplitude at point *P*_0_, *θ* is the angle between *P*_0_*P*_1_ and the *z* axis, *r* is the distance between *P*_1_ and *P*_*0*_, and *Σ* represents the integral domain of the mask pattern. According to the geometric relationship in Fig. 1, we can get

2 $$ r=\sqrt{{\left({x}_1-{x}_0\right)}^2+{\left({y}_1-{y}_0\right)}^2+{z_1}^2} $$

3 $$ \cos \theta ={z}_1/r $$

*E*(*P*_0_) is a constant in the model. Thus, the calculating formula becomes:

4 $$ E\left({P}_1\right)=\frac{E\left({P}_0\right)}{2 j\lambda}\underset{\sum }{\iint}\frac{\exp \Big( jk\sqrt{{\left({x}_1-{x}_0\right)}^2+{\left({y}_1-{y}_0\right)}^2+{z_1}^2\Big)}}{\sqrt{{\left({x}_1-{x}_0\right)}^2+{\left({y}_1-{y}_0\right)}^2+{z_1}^2}}\left(1+\frac{z_1}{\sqrt{{\left({x}_1-{x}_0\right)}^2+{\left({y}_1-{y}_0\right)}^2+{z_1}^2}}\right){dx}_0{dy}_0 $$

Then, the integrals are calculated using Matlab software, and the light intensity distribution in the photoresist can be expressed as:

5 $$ I\left(x,y,z\right)={\left|E\left({P}_1\right)\right|}^2 $$

where (*x*, *y*, *z*) equals the coordinate of *P*_1_.

In order to further investigate the absorption of the photoresist, we modified the calculations of the light intensity when considering the absorption coefficient. When a light beam passes through the photoresist from *P*_0_ to *P*_1_, the light intensity can be calculated by the following formula [25].

6 $$ \frac{I_{\alpha }}{I_0}=\exp \left(-\alpha r\right) $$

where *I*_0_ is the initial light intensity at point *P*_0_, *I*_*α*_ is the light intensity at point *P*_1_, *α* is the absorption coefficient of the photoresist, and *r* is the distance between *P*_0_ and *P*_1_. We define *I*_*α =* 0_ as the light intensity at point *P*_1_ when *α* = 0 μm^−1^. It is easy to obtain that *I*_*α =* 0_ = *I*_0_ according to formula (6). The relations between *E*(*P*_*α =* 0_) (the amplitude corresponding to *I*_*α =* 0_) and *E*(*P*_*α*_) (the amplitude corresponding to *I*_*α*_) can be expressed by:

7 $$ \frac{E\left({P}_{\alpha}\right)}{E\left({P}_{\alpha =0}\right)}=\exp \left(-\alpha r/2\right) $$

Thus, when considering the absorption of the photoresist in the diffraction lithography, the amplitude at point *P*_1_ (defined as *E*(*P*_1α_)) can be calculated by:

8 $$ E\left({P}_{1\alpha}\right)=\frac{1}{2 j\lambda}\underset{\sum }{\iint}\exp \left(-\alpha r/2\right)E\left({P}_0\right)\frac{\exp (jkr)}{r}\left(1+\cos \theta \right) ds $$

And the light intensity can be obtained by formulas (2), (3), (5), and (8).

### Experimental Details

**Fig. 2:**
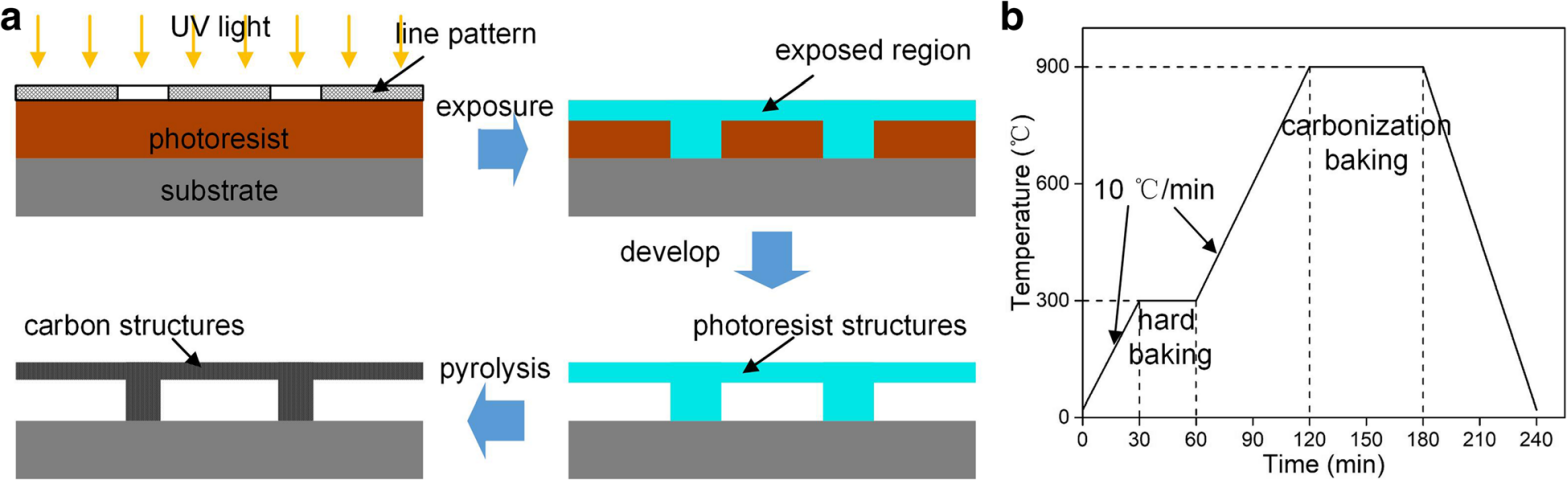
**a** The process for fabricating 3D carbon suspended structures. **b** The temperature curve of the pyrolysis

Masks with line patterns were used to fabricate suspended structures, while circles or squares were designed for fabricating pillars to support the suspended layer. Two kinds of thick negative photoresist were employed in the experiments, including SU-8 2100 (Microchem Co., Ltd.) with thickness of ~ 50 μm and NR26-25000P (Futurrex Co., Ltd.) with thickness of ~ 30 μm. The exposure process was performed with a MJB4 mask aligner, where the wavelength of the illuminating UV light was 365 nm. The suspended structures can be obtained after the samples were immersed into the developer for a certain time. Here, propylene glycol methyl ether acetate (PGMEA, Aladdin Co., Ltd.) was used as the developer for the SU-8 2100 samples and RD6 developer (Futurrex Co., Ltd.) was chosen for the NR26-25000P samples. Finally, a pyrolysis process [16, 26, 27] containing a hard baking step and a carbonization baking step was carried out in a quartz furnace (MTI GAL 1400X) to obtain 3D carbon microstructures. The whole process is illustrated in Fig. 2a, and the temperature variations during the pyrolysis process are illustrated in Fig. 2b. The samples were hard baked at 300 °C for 30 min and then pyrolyzed at 900 °C for 60 min. During the pyrolysis process, the samples were kept in the H_2_(5%)/Ar(95%) atmosphere with a heating rate of 10 °C/min. The obtained microstructures were characterized by a scanning electron microscope (SEM, Helios NanoLab G3, FEI).

## Results and Discussions

### Light Intensity Distribution

**Fig. 3:**
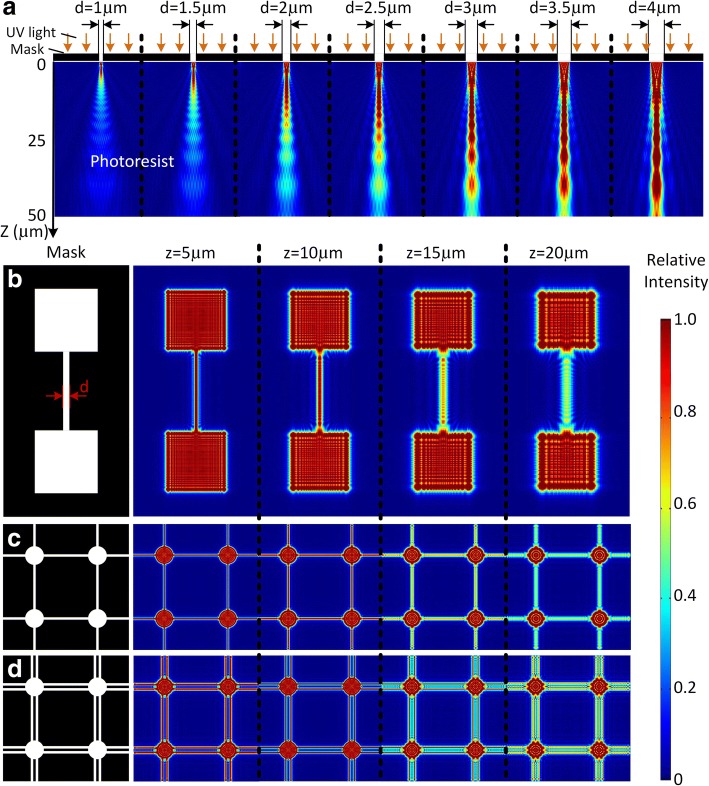
The mask patterns and simulation results in the photoresist. **a** The light intensity distributions below the photo mask under *d* = 1 μm, 1.5 μm, 2 μm, 2.5 μm, 3 μm, 3.5 μm, and 4 μm, where *d* is the width of the line pattern. The mask pattern for **b** suspended beams, **c** meshes, and **d** meshes with stacked line patterns and the corresponding light intensity distributions under *z* = 5 μm, 10 μm, 15 μm, and 20 μm in the photoresist. Here, *z* is the distance between the section plane and photo mask

Figure 3a shows the cross section of the 3D light intensity distribution under a line-shaped mask with the line width *d* = 1 μm, 1.5 μm, 2 μm, 2.5 μm, 3 μm, 3.5 μm, and 4 μm, respectively. Here, relative intensity is adopted, and the incident light intensity is defined as 1. The light at the bottom of the photoresist will gradually scatter owing to the light diffraction effect. Once the light intensity reaches a threshold value, the photoresist will get enough energy to release the reaction and turn solid; otherwise, it will be removed in the development process. The thickness of the region above the threshold (0.75 in this study) is defined as the exposure depth, which is very sensitive to the pattern width. The exposure depth is 5.3 μm under *d* = 1 μm and 18.2 μm under *d* = 2 μm. It will further increase to 33.5 μm under *d* = 3 μm and 47.5 μm under *d* = 4 μm. If the line width is narrower than 1 μm, the exposure depth will be too small for the fabrication, because the air gap between the mask and photoresist caused by the unevenness of the thick photoresist will make the exposure fail. Figure 3b, c shows the mask patterns for fabricating suspended structures and the corresponding light intensity distribution at *z* = 5, 10, 15, and 20 μm, where the line width is set as 2 μm. The exposure depth of the line and mesh patterns is between 15 and 20 μm, while that of the large squares and circles is big enough to form pillars during lithography. Thus, suspended beams and meshes can be fabricated, supported by the pillars. Since it is hard to fabricate suspended structures when the line width is greater than 5 μm, line patterns are stacked side by side to fabricate wide suspended beams or meshes, as shown in Fig. 3d.

### Suspended Photoresist Structures

**Fig. 4:**
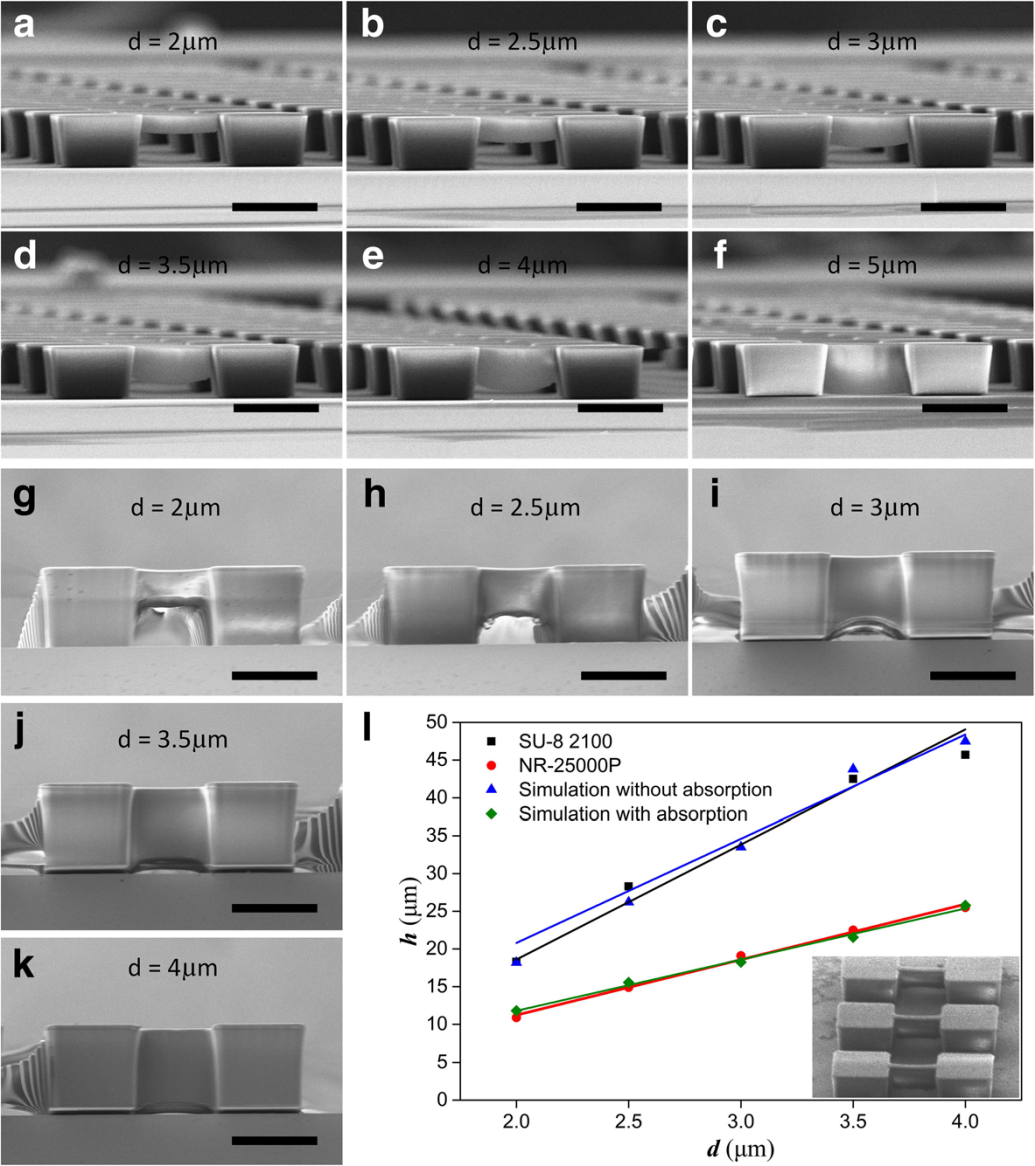
The suspended photoresist beams resulted from one-step diffraction lithography with different line width *d* using the mask pattern in Fig. 3b. NR26-25000P photoresist beams under **a** *d* = 2 μm, **b** 2.5 μm, **c** 3 μm, **d** 3.5 μm, **e** 4 μm, and **f** 5 μm; SU-8 2100 photoresist beams under **g** *d* = 2 μm, **h** 2.5 μm, **i** 3 μm, **j** 3.5 μm, and **k** 4 μm; **l** the functions of exposure thickness vs. line width in simulation without absorption, NR26-25000P, and SU-8 2100 and simulations with absorption coefficient *α* = 0.0374 μm^−1^, where the inset shows the tilted view of SU-8 suspended beams. The thickness of the beams increases with the line width of the mask pattern. The scale bars are 50 μm

Experiments were carried out to fabricate suspended structures. We tested the minimum exposure time to obtain photoresist pillars and defined it as the exposure threshold. Then, four or three times of the threshold value was adopted as the exposure dose and the threshold of the relative light intensity was evaluated at 0.75, in accordance with the simulation. Figure 4 shows the suspended photoresist beams under different *d* value. It is found that the thickness of the suspended layer *h* is positively related to *d*. For NR26-25000P photoresist, *h* is 10.9 μm under *d* = 2 μm (Fig. 4a) and increases to 25.5 μm under *d* = 4 μm (Fig. 4e). As *d* comes to 5 μm, the exposure depth is big enough to reach the substrate, and no suspended structures is obtained (Fig. 4f). Figure 4g–k depicts the suspended structures of SU-8. The function of *h* vs. *d* for both experiments and simulations is illustrated in Fig. 4l, where the straight lines are fitted by the least square method. The linear correlation coefficient *R* of the fitted lines are *R*^2^ = 0.963, 0.988, and 0.858 for simulations without counting the absorption, NR26-25000P, and SU-8, respectively. It can be seen that the results of the SU-8 experiments are very close to the simulation results. By contrast, the suspended layer of NR26-25000P is much thinner than that of the simulation without absorption. This can be mainly attributed to the transparent property of SU-8 and the high absorption ability of NR26-25000P. This is also why gray exposure can be used to fabricate suspended structures for some photoresist, but not suitable for SU-8.

Then, we introduce absorption coefficient *α* in optical model and perform the calculations with formula (8). The results under *α* = 0.0374 μm^−1^ (the absorption coefficient of NR21-25000P at 365 nm, tested by a UV-visible spectrophotometer, UV 2600, Shimadzu Co., Ltd.) are shown in Fig. 4l, where the fitted line with *R*^2^ = 0.986 agrees well with the experimental results of NR26-25000P. Thus, our method is available for almost all kinds of thick negative photoresist to fabricate suspended structures with one-step exposure, in which the exposure depth can be guided through simulations.

**Fig. 5:**
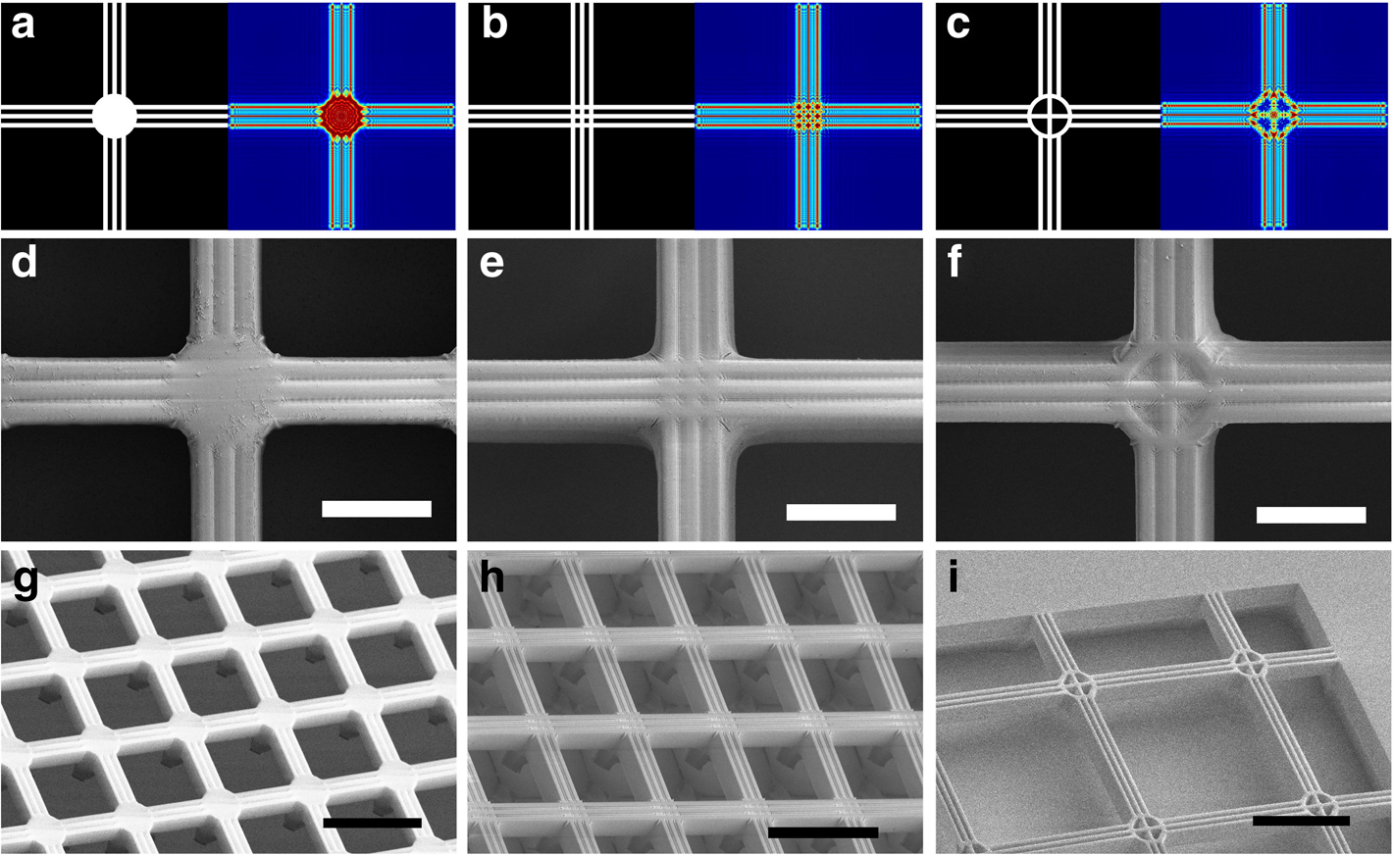
Different cross connection patterns with NR26-25000P. **a**–**c** Three cross connection patterns on the mask and the corresponding simulation results at *z* = 15 μm, where the line width is 2 μm with spacing of 2 μm and *z* is the distance between the section plane and photo mask. **d**–**f** The textures on the obtained photoresist cross connection and the broad beams, where the scale bars are 20 μm. **g** The suspended meshes with supporting pillars. **h** The suspended meshes with thin supporting pillars, where the pillars result from the dense cross connection patterns with high ratio. **i** The full suspended mesh patterns. The scale bars in **g**–**i** are 100 μm

Figure 5a–c displays the varied cross connection patterns and the corresponding simulation results at *z* = 15 μm. Three lines are stacked side by side to fabricate a broad suspended beam, where the line width and interval width are both 2 μm. The cross connection pattern with a 20-μm circle is used to fabricate a pillar to support the suspended beams (Fig. 5a). Hollow cross connection patterns are designed to fabricate suspended meshes, as exhibited in Fig. 5b, c. The obtained NR26-25000P photoresist connections are shown in Fig. 5d–f, where the surface textures on the cross connections together with the beams can be clearly observed, in good agreement with the simulations (Fig. 5a–c). Suspended meshes with the three types of cross connections are shown in Fig. 5g–i, and the supporting pillars are also obtained as expected (Fig. 5g). Figure 5h illustrates the thin pillars under the cross connections, owing to the dense patterns with high ratio. The cross connection pattern in Fig. 5c possesses lower duty ratio, where the light intensity is weak, resulting in a full suspended mesh (Fig. 5f). Thus, the ratio of the cross connection patterns can be reduced to fabricate full suspended structures, while the supporting pillars can be easily formed with a solid connection. Meanwhile, the width of the beam can also be controlled by adjusting the number of the stacked line patterns.

**Fig. 6:**
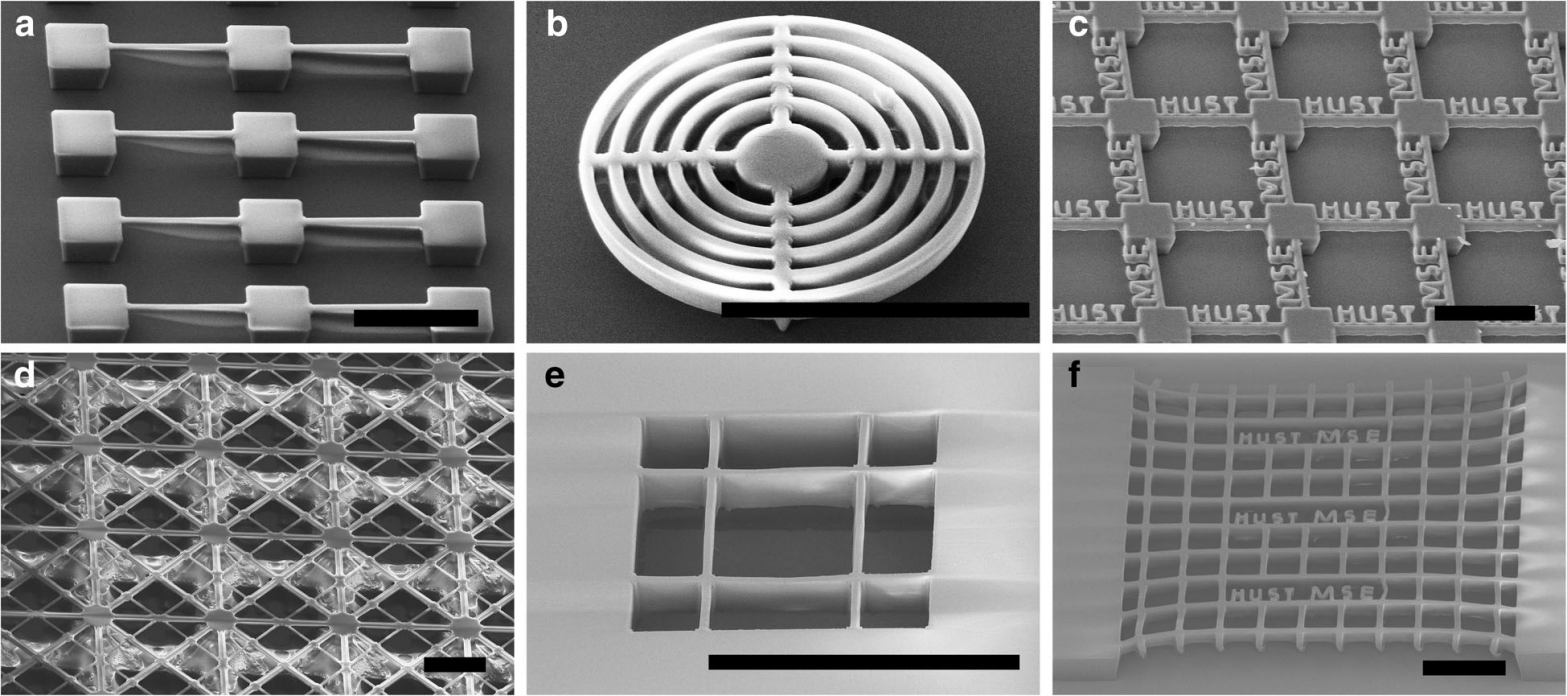
3D suspended photoresist structures. **a** Suspended beams with gradient thickness, **b** suspended concentric rings, **c** suspended word structures, and **d** multilayer suspended meshes, where the photoresist is NR26-25000P. **e** Suspended SU-8 mesh. **f** Suspended SU-8 meshes with word patterns. The scale bars are 100 μm. The suspended structures in **d** is achieved by a two-step exposure, and the others are fabricated with a one-step exposure

Some complex 3D microstructures have also been fabricated via a single exposure (Fig. 6a–c, e, f) or a two-step exposure (Fig. 6d) method. Suspended beams with gradient thickness are shown in Fig. 6a, where the width of the line patterns varies from 2 to 4 μm and 4 to 6 μm in the two regions. The thickness of the suspended layer increases with the increase of the line width, in line with the results displayed in Fig. 4. Suspended concentric rings and suspended word patterns can also be easily prepared (Fig. 6b, c). By combining the two exposure processes, two suspended layers have been integrated with NR26-25000P, as shown in Fig. 6d. After the first exposure is completed, the second layer is then spin-coated on the first layer and exposed. The stacked meshes are achieved after the two exposure processes followed by a developing process. Since the second exposure may cause damage to the first layer, the structures need to be carefully optimized to fabricate more excellent multi-layer suspended structures. SU-8 photoresist suspended meshes with word patterns have also been successfully achieved (Fig. 6d–f), though it is more difficult than NR26-25000P to control the exposure parameters due to the high transparency.

Compared with previous works [2, 11, 22, 23], we form a 3D light intensity distribution model in the photoresist by utilizing the diffraction of the small mask patterns. The 3D suspended structures can be well controlled and forecasted by simulations. The absorption coefficient of the photoresist is also taken into account here. Suspended structures with various thicknesses, such as gradient beams, are formed easily through the one-step exposure. Moreover, the exposure process is performed with an ordinary mask in a typical contact exposure mode, and no special masks or equipment is needed, exhibiting excellent compatibility with high fabrication resolution.

### Pyrolytic Carbon Structures

**Fig. 7:**
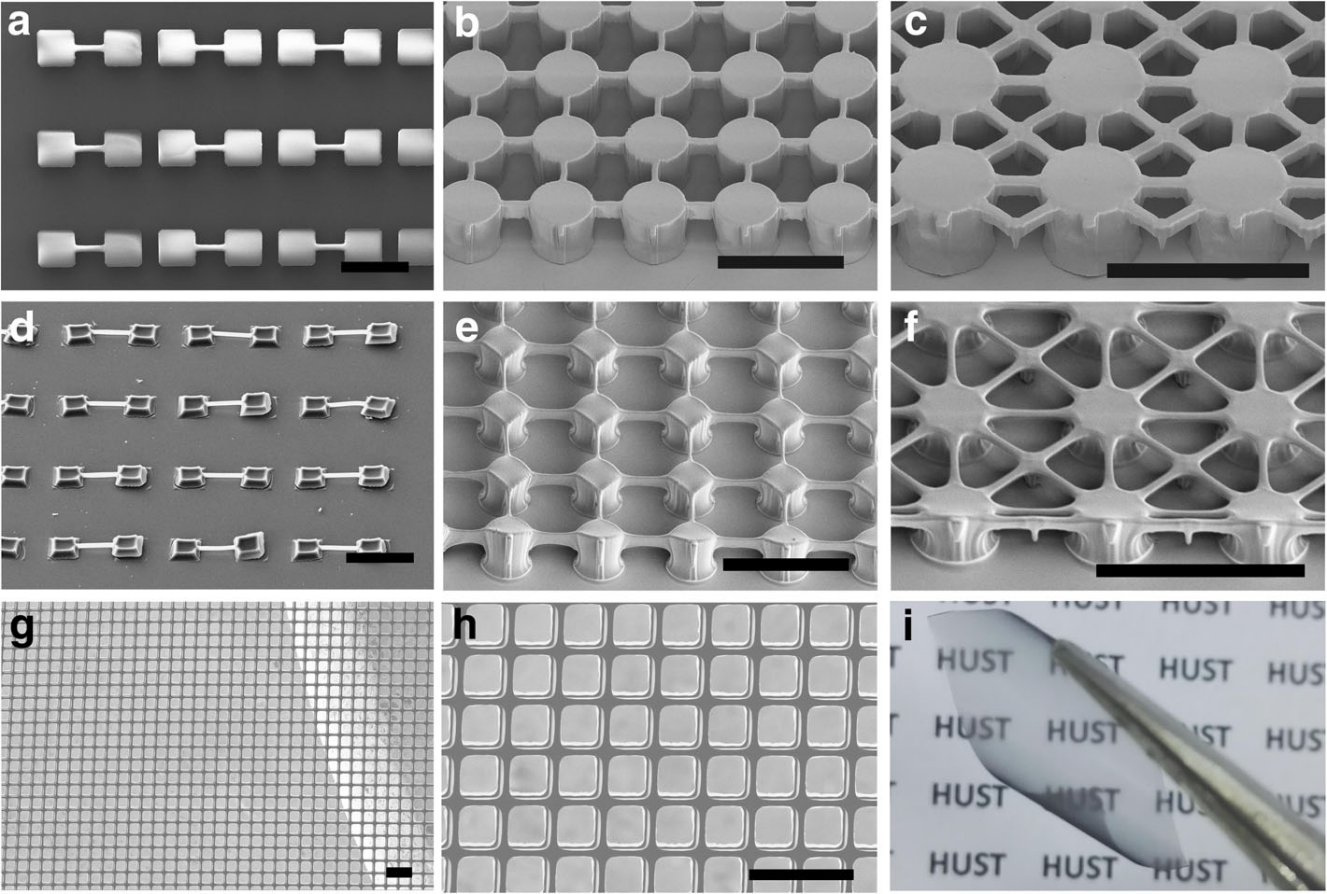
Suspended SU-8 meshes and pyrolytic carbon meshes. **a** Suspended SU-8 beams. **b**, **c** Suspended SU-8 meshes with supporting pillars. **d** Suspended carbon beams, where great strains remained in the carbon structures and cracks occurred at the bottom of the pillar. **e**, **f** Suspended carbon meshes. **g** Free-standing carbon mesh after pyrolysis. **h** Magnification of the free-standing carbon mesh. **i** A 12 mm × 20 mm free-standing carbon mesh, which presents well flexibility and transparency. The scale bars are 100 μm

SU-8 is a typical precursor for the fabrication of carbon microstructures, while other photoresists like NR26-25000P are not able to sustain the structures under high temperature. Figure 7a–c shows the suspended SU-8 structures while the corresponding pyrolytic carbon structures are presented in Fig. 7d–f. Large shrinkage occurs during the pyrolysis process owing to the multiple concurrent reactions, including dehydrogenation, cyclization, condensation, hydrogen transfer, and isomerization [8, 28]. Thus, a considerable residual stress will exist in the pyrolytic structures, especially in the asymmetric structures. The pyrolytic carbon beams will shrink and pull the pillars at both ends, causing cracks at the bottom (Fig. 7d). As for the large-scale meshes, the stress maintains a relative balance in each direction and no obvious cracks are found in the pyrolytic carbon structures (Fig. 7e, f). Free-standing carbon meshes with the size of 12 mm × 20 mm are achieved, as shown in Fig. 7g–i. The sheet resistance of the carbon meshes is about 182 Ω/sq, and the light transmittance reaches ~ 67% in the whole wavelength. The as-prepared carbon meshes with superior conductivity and transparency can be applied into perovskite solar cells as electrode [29–31], offering an available method for fabricating semitransparent solar cells. Moreover, the as-prepared carbon meshes possess excellent flexibility, demonstrating great potential in the applications of flexible transparent electrodes.

## Conclusions

In summary, we demonstrated the fabrication of suspended structures via a novel one-step mask-based diffraction lithography method. The 3D light intensity distribution in the photoresist was simulated, showing that the exposure depth increased with the increase of the width of the line patterns under *d* < 5 μm. This phenomenon could be utilized to fabricate suspended structures with defined thickness of SU-8 photoresist, which was almost transparent and hard to form suspended structures with grayscale lithography. The corresponding experiments were also conducted here. We found that the thickness of the suspended SU-8 beams was very close to the simulation results, while that of the NR26-25000P was much thinner than the exposure depth in the simulations. This was caused by the high light absorption property of NR26-25000P. Then, the absorption coefficient of photoresist was introduced in the optical model, and the simulation results agreed well with the experiments. Three different cross connection patterns were designed for fabricating suspended 3D meshes with or without supporting pillars, and the surface textures were well replicated. Meshes with pillars and full suspended meshes were also successfully achieved. Other complex 3D suspended photoresist structures, including suspended beams with gradient thickness, suspended concentric rings, and suspended word structures, were obtained through the one-step mask-based diffraction lithography.

Carbon suspended structures and free-standing carbon meshes were further fabricated with a typical two-step pyrolysis process. The suspended 3D carbon structures could be applied in electrochemical electrode, supercapacitor, and sensors owing to their large surface area. The free-standing meshes exhibited excellent conductivity, flexibility, and high transparency. Thus, we developed a simplified and promising method for the fabrications of 3D suspended structures and carbon meshes, which showed great potential in the applications of transparent electrode, semitransparent solar cells, and energy storage devices.

## Abbreviations

3D: Three-dimensional
C-MEMS: Carbon microelectromechanical systems
